# Late infantile metachromatic leukodystrophy: Clinical manifestations of five Taiwanese patients and Genetic features in Asia

**DOI:** 10.1186/s13023-015-0363-1

**Published:** 2015-11-09

**Authors:** Hsiang-Ru Liaw, Hsiu-Fen Lee, Ching-Shiang Chi, Chi-Ren Tsai

**Affiliations:** Department of Pediatrics, Tungs’ Taichung Metroharbor Hospital, 699, Taiwan Boulevard Sec. 8, Wuchi, Taichung, 435 Taiwan; Department of Pediatrics, Taichung Veterans General Hospital, 1650, Taiwan Boulevard Sec. 4, Taichung, 40705 Taiwan; School of Medicine, Chung Shan Medical University, 110, Sec. 1, Jianguo N. Rd, Taichung, 40201 Taiwan; Institute of Molecular Biology, National Chung Hsing University, 250, Kuo Kuang Rd, Taichung, 402 Taiwan

**Keywords:** Metachromatic leukodystrophy, ARSA gene mutation, Taiwan, Asia

## Abstract

**Background:**

This study was conducted to describe the clinical and genetic features of patients with late infantile metachromatic leukodystrophy.

**Methods:**

Clinical and genetic manifestations of five Taiwanese patients with late infantile metachromatic leukodystrophy from January 2003 to April 2014 were reviewed. The genetic features of such patients reported in Asian countries during a period of 20 years were also analyzed.

**Results:**

The median age at disease onset was 1 year and 3 months with the first clinical symptom being gait disturbance. All five patients became bed-ridden at a median age of 2 years and 5 months. Nerve conduction velocity revealed demyelinating polyneuropathy and brain MRI disclosed tigroid and leopard skin pattern of dysmyelination in all 5 patients. All patients had decreased ARSA activities in leukocytes accounting for 15.88 % to 30.75 % of controls. Five novel mutations, p.A316D, p.G303R, p.Q176X, p.R293X, and c.749 insGCGGGCCA, were identified in our case series. Eighteen patients, including our 5 patients, were reported in Asian countries. A total of 22 different disease-causing alleles were found, in which p.W320X was identified in Taiwan and China, and p.G101V was found in Taiwan and Korea.

**Conclusions:**

Patients with late infantile metachromatic leukodystrophy exhibited a rapid and devastating clinical course. The pattern of dysmyelination on brain MRI together with peripheral demyelination polyneuropathy indicates that evaluation of ARSA activity in leukocytes is warranted. A wide diversity of ARSA gene mutations was noted in Asia.

## Background

Metachromatic leukodystrophy (MLD) is a rare autosomal recessive inherited disease, which is caused by a deficiency in the enzyme activity of Arylsulfatase A (ARSA). ARSA is required for the hydrolysis of sulfated glycosphingolipids, which are also known as sulfatides, and its deficiency results in excessive accumulation of sulfatide in myelin in the nervous system, the bile ducts of the liver, and the distal tubules of the kidney [1–3]. ARSA deficiency results from a mutation in the ARSA gene, which spans 3.2 kb of genomic DNA on chromosome 22q13 [2, 4]. Clinically, the differences in residual enzyme activity of ARSA cause a great diversity of onset age of the disease and the severity of the clinical course. Based on the age of disease onset, MLD can be divided into three forms: late infantile, juvenile, and adult MLD [4].

Late infantile MLD is the most common form of MLD, which accounts for 50–60 % of all cases [5] and incidence is estimated to range from 1 in 40,000 to 1 in 170,000 newborns [6]. The age of disease onset is usually between 18 and 24 months with the first recognizable feature of gait disturbance. Afterwards, rapid deterioration of motor, speech, and intellectual functions develop over the following months [7, 8]. Because of the profound ARSA deficiency and progressive demyelination, such cases usually manifest a rapid and devastating neurodegenerative clinical course [1]. Inevitable neurological sequelae develop as the disease progresses, such as decorticate postures, impaired feeding and swallowing due to pseudobulbar palsies, seizures, and severe psychomotor retardation. Those patients eventually expire within the first decade of life [4]. Currently, feasible therapeutic options are limited to palliative and supportive treatments [1, 2, 9–12].

Here we report the clinical manifestations, neuroimaging studies, ARSA enzyme activity, ARSA gene mutations, and neurological outcomes of five Taiwanese patients with late infantile MLD. In addition, the differences in ARSA gene mutations between Taiwan and other Asian countries are noted.

## Methods

### Study sample

This was a retrospective, uncontrolled, nonblinded study in which cases from a period spanning over a decade were analyzed. Between January 2003 and April 2014, a total of five patients diagnosed with late infantile MLD were enrolled. All patients underwent detailed physical and neurological examinations, and basic laboratory tests, including complete blood count, blood sugar, creatine phosphokinase, electrolytes, liver function test, renal function test, blood lactate level, arterial blood gas analysis, and urine routine, as well as metabolic surveys, including assays of blood amino acids and urinary organic acids. Spinal tap was administered to patients with their parents’ consent, and the values of cerebrospinal fluid (CSF) and assay of CSF amino acids were determined. Neurophysiological studies, including electroencephalography (EEG), auditory evoked potential (AEP), and visual evoked potential (VEP) were performed. Biochemical enzyme activities for lysosomal disorders were analyzed. Brain magnetic resonance imaging (MRI) and nerve conduction velocity (NCV) were performed with their parents’ consent.

The diagnostic criteria of late infantile MLD in our case series included: 1) disease onset at age younger than 2 years following by a clinically devastating regression of motor, language, and cognition functions; 2) tigroid and leopard skin pattern of dysmyelination on the brain MRI; 3) decreased ARSA activity in leukocytes; and 4) pathognomonic ARSA gene mutations [4].

### Molecular analysis

Genomic DNA was extracted from peripheral blood samples. All the exons of the ARSA gene were amplified by polymerase chain reaction (PCR) with their corresponding intronic primers. PCR products were subjected to bidirectional sequencing using a Big-Dye Terminator v3.1 Cycle Sequencing Kit and an ABI Prism 3100 genetic analyzer (Applied Biosystems, Foster City, CA). The protein sequence was based on GenBank accession no. NM_00487.5. Written informed consent was obtained from all participants’ parents prior to collection of blood samples for molecular studies.

### Data collection

The medical records of these five patients were reviewed. Patients’ clinical information, including gender, age at disease onset, histories of developmental milestones prior to the onset of the disease, duration between disease onset and psychomotor regression, and clinical outcomes, were collected. Laboratory data, neuroimaging findings, neurophysiological studies, ARSA enzyme activities, and ARSA gene mutations were analyzed. This study was approved by the Institutional Review Board (IRB TCVGH No. CF14213).

### Literature review

We searched the MEDLINE and PubMed database using the keywords “metachromatic leukodystrophy”. We reviewed cases diagnosed with late infantile MLD and reported in Asian countries from the period 1995 to 2014. In our study, patients with molecular analysis of ARSA gene mutations were recruited. In order to make meaningful comparisons between ARSA gene mutations in Taiwan and those reported in other Asian countries, the genomic data of all patients identified in the MEDLINE and PubMed search were re-interpreted with respect to the change in protein sequences of ARSA amino acids based on GenBank accession no. NM_00487.5 if a patient’s sequencing results were originally interpreted by GenBank accession no. NM_00487.3 or GenBank accession no. NM_00487.4.

## Results

The clinical features, laboratory data, neurophysiological studies, and neuroimaging findings of the five late infantile MLD Taiwanese patients, three boys and two girls, are shown in Table 1.

**Table 1:** Clinical manifestations, laboratory data, neurophysiological studies, and neuroimaging findings of five patients with late-infantile metachromatic leukodystrophy

Patient	1	2	3	4	5
Gender	M	F	F	M	M
Age at disease onset	1 yr 3mo	1 yr 2mo	1 yr 3mo	1 yr 2mo	1 yr 11mo
Age at first visit	2 yr 4mo	1 yr 11mo	2 yr	1 yr 8mo	2 yr
Neurological examinations at first visit					
Muscle tone	Hypertonicity	Hypertonicity	Hypertonicity	Hypertonicity	Hypertonicity
Deep tendon reflexes	Increased	Increased	Increased	Increased	Increased
Babinski sign	Negative	Positive	Positive	Positive	Positive
Ankle clonus	Negative	Negative	Positive	Negative	Positive
Posturing	Spasticity	Spasticity	Decorticate	Spasticity	Spasticity
Laboratory data					
CSF protein level, mg/dl (Normal range, 20-45 mg/dl)	Not done	135	200	171.4	Not done
Neurophysiological studies					
Electroencephalography					
At first visit	AS	AS	AS	AS	AS
Follow-up	Not done	AS	AS, BS	BS, focal spikes	BS
Auditory evoked potential	No response	Normal	Normal	No response	Not done
Visual evoked potential	Right side delay	Normal	Normal	Normal	Not done
Nerve conduction velocity					
Demyelinating polyneuropathy	Yes	Yes	Yes	Yes	Yes
Neuroimaging findings					
Brain MRI					
Dysmyelination pattern resembling tiger skin	Yes	Yes	Yes	Yes	Yes
Spinal MRI					
Signal change over the white matter	Not done	Yes	Yes	Not done	Not done
Psychomotor regression					
Bed-ridden status, age	2 yr 5mo	2 yr 2mo	3 yr 4mo	1 yr 7mo	3 yr 6mo
Being unable to speak, age	Not available	2 yr	3 yr	1 yr 7mo	3 yr
Loss of eye contact, age	Not available	Not available	3 yr 5mo	2 yr 9mo	4 yr 4mo
Seizure onset, age	Not available	Not available	3 yr	Never	3 yr 6mo
Neurological follow-up					
Gastric tube implantation for feeding, age	Not available	2 yr 6mo	3 yr 8mo	2 yr	3 yr 6mo
Home BiPAP for respiratory support, age	Not available	5 yr 1mo	Nil	Nil	Nil
Outcome	Loss of follow-up	Died	Loss of follow-up	Died	Alive
		7 yr 4mo		4 yr 9mo	8 yr

*AS* absence of sleep spindles, *BS* background slowing, *CSF* cerebrospinal fluid, *F* female, *M* male, *mo* months, *MRI* magnetic resonance imaging, *yr* years

The median age at disease onset was 1 year and 3 months, with a range from 1 year 2 months to 1 year and 11 months. All of the patients exhibited the first recognizable clinical symptoms of gait disturbances with brisk deep tendon reflexes and increased tonicity over the bilateral lower legs upon neurological examinations at the first visit. After the onset of the disease, rapid psychomotor regression developed. All patients became bed-ridden at a median age of 2 years and 5 months, ranging from 1 year 7 months to 3 years and 6 months. The times at which language function regressed to “unable to speak” and social function deteriorated to “loss of eye contact” were available in 4 and 3 patients, respectively. The median age of symptoms in the aforementioned patients was 2 years and 6 months and 3 years and 5 months, respectively. Epileptic seizures occurred in patients 3 and 5 at age 3 years and 3 years and 6 months, respectively. Regarding the clinical outcomes during the long-term period of follow-up, patients 2 and 4 died of respiratory failure at the ages of 7 years and 4 months and 4 years and 9 months, respectively. Patient 5, aged 8 years, is still alive with significant neurological sequelae. Patients 1 and 3 were lost to follow-up.

**Fig. 1:**
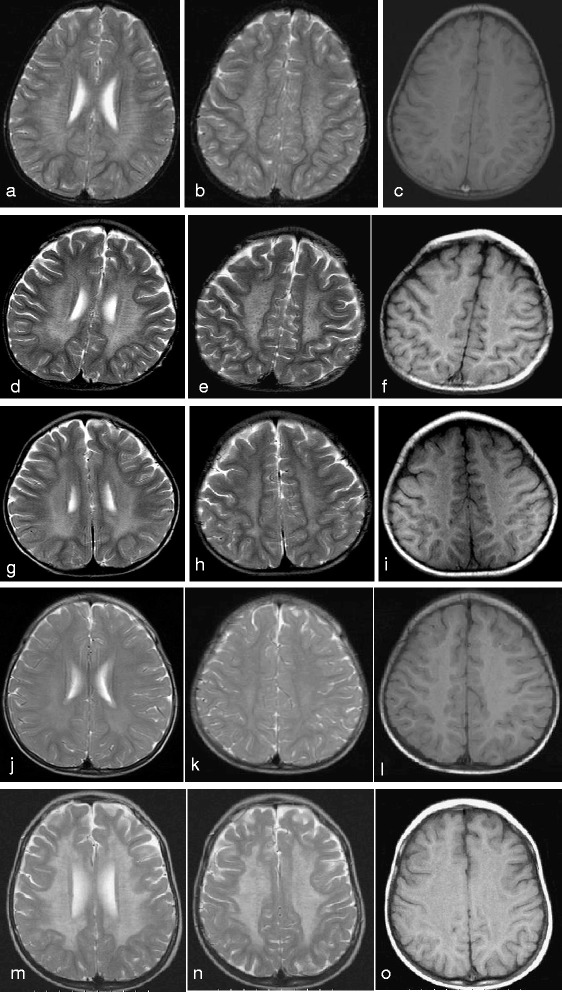
Cranial MRIs of our patients. **a**-**c** represents patient 1, **d**-**f** patient 2, **g**-**i** patient 3, **j**-**l** patient 4, and **m**-**o** patient 5. (**a**, **d**, **g**, **j**, **m**) Hypointense radially oriented stripes and dots seen within the hyperintense cerebral white matter (resembling tiger skin) on T2-weighted axial imaging. (**b**, **e**, **h**, **k**, **n**) Hypointense dots resembling leopard skin seen on T2-weighted axial imaging at the level of centrum ovale. (**c**, **f**, **i**, **l**, **o**) Iso to hyperintense dots seen in the cerebral white matter on T1-weighted imaging. This pattern of dysmyelination resembles the skin of tiger (radial stripes) and leopard (dots), the so-called tigroid and leopard pattern of dysmyelination in metachromatic leukodystrophy

Three patients received a spinal tap and all of them showed elevated levels of protein in CSF analysis. The results of neurophysiological studies revealed that patients 1 and 4 showed no response with respect to AEPs and patient 1 had a delayed response on the VEP evaluation. EEG at the first visit revealed nonspecific findings with absence of sleep spindles in all five patients. The follow-up EEGs were recorded depending on the patients’ clinical condition: EEGs showed nonspecific findings with background slowing in three patients and focal spikes in patient 4. NCV and brain MRI were performed in all five patients. NCVs revealed demyelinating polyneuropathy. Cranial MRIs disclosed tigroid and leopard skin pattern of dysmyelination (Fig. 1a-o). Spinal MRIs of patients 2 and 3 showed signal changes over the white matter.

**Fig. 2:**
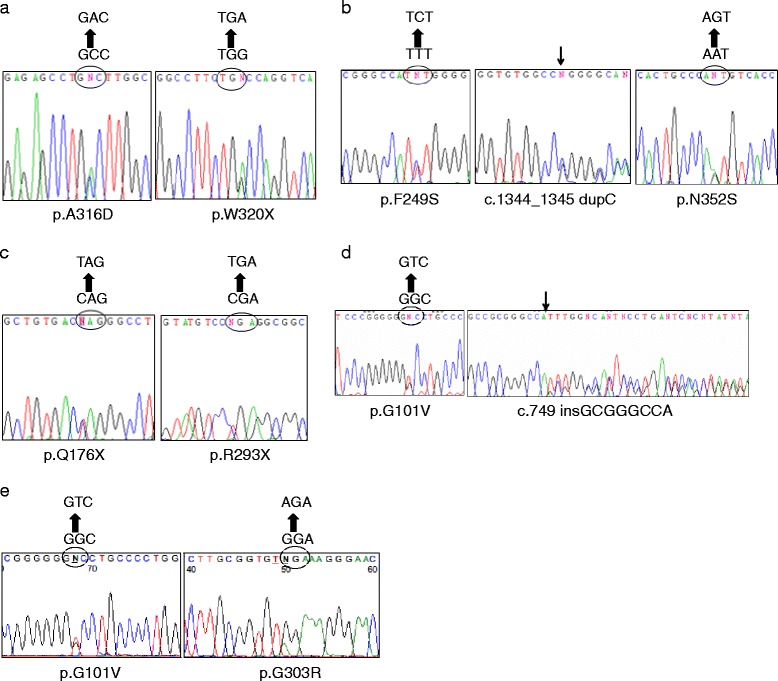
ARSA gene mutations of our patients. **a** Patient 1 has ARSA mutations of p.A316D and p.W320X. **b** Patient 2 has ARSA mutations of p.F249S, c.1344_1345 dupC, and an additional pseudodeficiency allele of p.N352S. **c** Patient 3 has ARSA mutations of p.Q176X and p.R293X. **d** Patient 4 has ARSA mutations of p.G101V and c.749 insGCGGGCCA. **e** Patient 5 has ARSA mutations of p.G101V and p.G303R

ARSA activity in leukocytes and ARSA gene mutations in our case series are shown in Table 2. All cases had significantly decreased ARSA activities in leukocytes with levels of 11.29 to 21.86 nmol/per milligram of protein/hr (normal, > 71 nmol/per milligram of protein/hr), which accounted for 15.88–30.75 % of controls. For the ARSA gene mutations, a total of eleven alleles were identified, including an additional pseudodeficiency allele of p.N352S in patient 2 (Fig. 2a-e). There were nine different disease-causing alleles, including four missense mutations of p.A316D, p.F249S, p.G101V, and p.G303R, three nonsense mutations of p.W320X, p.Q176X, and p.R293X, and two frameshift mutations of c.1344_1345 dupC and c.749 insGCGGGCCA. Five alleles, p.A316D, p.G303R, p.Q176X, p.R293X, and c.749 insGCGGGCCA, were novel mutations. All our reported cases were heterozygous gene mutations.

**Table 2:** ARSA enzyme activities and ARSA gene mutations in our case series and in reported cases in other Asian countries

Patient [Ref]	Age of dz onset	ARSA enzyme activity, nmol/mg Protein/hr (Normal reference)	ARSA enzyme activity, % of control range	ARSA gene mutation Protein sequence of ARSA amino acid change by different GenBank		
				GenBank accession no. NM_00487.3	GenBank accession no. NM_00487.4	GenBank accession no. NM_00487.5
Taiwan						
1	1 yr 3mo	11.30 (>71.1)	15.89	Not used	Not used	p.A316D ^a^/p.W320X
2	1 yr 2mo	11.81 (>71.1)	16.61	Not used	Not used	p.F249S/c.1344_1345 dupC p.N352S ^b^
3	1 yr 3mo	11.29 (>71.1)	15.88	Not used	Not used	p.Q176X ^a^ /p.R293X ^a^
4	1 yr 2mo	15.23 (>71.1)	21.42	Not used	Not used	p.G101V/c.749 insGCGGGCCA ^a^
5	1 yr 11mo	21.86 (>71.1)	30.75	Not used	Not used	p.G101V/p.G303R ^a^
India						
6 [13]	1 yr 6mo	1.23 (>50)	2.46	Not used	c.459 + 1G > A/not found	c.465 + 1G > A/not found
7 [13]	2 yr 4mo	2.43 (>50)	4.86	Not used	p.Y33S/not found	p.Y35S/not found
8 [13]	1 yr 6mo	2.45 (>50)	4.90	Not used	p.R311Q/ p.R311Q	p.R313Q/ p.R313Q
9 [13]	2 yr 3mo	Undetectable (>50)	NA	Not used	c.459 + 1G > A/ c.459 + 1G > A	c.465 + 1G > A/ c.465 + 1G > A
10 [13]	1 yr 6mo	0.83 (>50)	1.66	Not used	c.752_753insT/not found c.1524 + 95A > G ^b^	c.758_759insT/not found c.1530 + 95A > G ^b^
11 [13]	2 yr 6mo	1.40 (>50)	2.80	Not used	p.R390W/ p.R390W	p.R392W/ p.R392W
12 [13]	2 yr	5.00 (>50)	10.0	Not used	p.G245R/ p.G245R	p.G247R/ p.G247R
Japan						
13 [14]	NA	NA	NA	Unknown	Unknown	p.Q155H/ p.G310V
14 [15]	NA	NA	NA	Unknown	Unknown	p.L300S/ c.225 + 2A > G
15 [16]	1 yr 11mo	15.80 (109.0-217.2)	14.50	Not used	Not used	p.P138T/ p.P138T
China						
16 [17]	1 yr 5mo	7.00 (38.9-98.3)	17.99	p.W318X/ p.W318X	Not used	p.W320X/ p.W320X
17 [18]	1 yr 7mo	1.19 (NA)	NA	Not used	Not used	c.622delC/ c.622delC
Korea						
18 [19]	1 yr	4.92 (30–90)	16.40	Unknown	Unknown	p.G101V/c.1107 + 1G > T

*ARSA* arylsulfatase A, *mo* months, *NA* not available, *Ref* reference, *yr* years

^a^Novel ARSA gene mutations,^b^ARSA pseudodeficiency allele

As shown in Table 2, 13 patients with late infantile MLD in Asian countries were reported in the period 1995–2014 [13–19], including seven from India, three from Japan, two from China, and one from Korea. Based on GenBank accession no. NM_00487.5 for changes in protein sequences of ARSA amino acids, a wide diversity of ARSA gene mutations were found. Excluding 3 cases in India for whom 3 alleles were not found, 15 different alleles in 23 alleles were identified. Six of 13 patients carried heterozygous gene mutations and seven were homozygous ARSA gene mutations. Taken together, there was a total of 18 patients, including our 5 patients, diagnosed with late infantile MLD with ARSA gene mutations in Asia, and 22 different alleles were identified. p.W320X was found in Taiwan and China, and p.G101V in Taiwan and Korea. Both p.W320X and p.G101Vwere found in three of the 33 alleles, with allele frequency of 9 %.

## Discussion

The clinical course of patients with late infantile MLD typically involves initial presentation of unsteady gait at the age of 1 year and 6 months, according to reports in the literature. Around the age of 3 years, they no longer have any locomotion activity and head or trunk control is absent, ultimately leading to bed-ridden status [7, 20]. In addition to motor function regression, they also experience the first language difficulties at a median age of about 2.5 years, and loss of verbal communication occurs at about 2 to 2.5 years after the onset of the disease [8]. Overall, our case series also observed a similar clinical course to those reported in the literature report. The median durations between disease onset and motor function regression to bed-ridden status and no verbal communication were 1 year and 2 months, and 11.5 months, respectively. Regarding the clinical outcomes, there are no reliable treatment options for patients with late infantile MLD [1, 9–12]. With supportive treatment, including gastric tube placement for feeding and antibiotic coverage during infection, patients could survive in a vegetative state for years [20], but they often die of aspiration pneumonia or bronchopneumonia within the first decade of life [4]. Patients 2 and 4 in our series died of respiratory failure 6 years and 2 months and 3 years and seven months, respectively, after disease onset.

In patients with late infantile MLD, T2-weighted brain MRI usually showed symmetric high signal intensity initially in the parietal-occipital central white matter, followed by frontal central white matter changes, which then spread to the commissural fibers of the corpus callosum and the periventricular white matter with a homogeneous change of pattern parallel to the course of the disease [21–24]. In addition to central nervous system (CNS) involvement, peripheral neuropathy was also evident in these patients, which was associated with uniform slowing of both motor and sensory NCVs and risk of developing unsteady gait and ataxia [25–28]. All of our 5 patients had characteristic tigroid and leopard skin pattern of dysmyelination on brain MRI [29] and demyelination polyneuropathy on NCV. If there appears to be involvement in both the CNS and peripheral nerve system in patients with suspected late infantile MLD, this could provide a valuable diagnostic clue for clinical pediatric neurologists to proceed to the next step and evaluate ARSA activity in leukocytes [30].

A pathogenic factor of late infantile MLD is absence or dramatic loss of ARSA activity, which usually accounts for less than 15 % of controls [31]. Our case series showed the reduction in ARSA activity ranged from 15.88–30.75 % compared with controls. However, low ARSA level was not sufficient to make a definitive diagnosis of MLD. In healthy individuals, some people have ARSA activity as low as 15–50 % of controls. This condition is called ARSA pseudodeficiency, which is a polymorphism [31]. Although a slight deficiency in ARSA activity, with levels 50–70 % that of controls, might be associated with pervasive developmental disorders, ARSA pseudodeficiency does not lead to neurological impairments [31]. In the general population, the frequency of ARSA pseudodeficiency alleles is about 7–15 %. Therefore, an accurate diagnosis of late infantile MLD is based on a combination of clinical features, biochemical analysis of low residual enzyme activity of ARSA in leukocytes, together with pathognomonic ARSA gene mutations [32]. Regarding the correlation between ARSA activity and age of disease onset, we observed that our patients 1 to 4 had lower ARSA activities, ranged from 11.29 to 15.23 nmol/mg Protein/hr, whose disease onset were between 1 year 2 months old and 1 year 3 months old. Patient 5 had ARSA activity of 21.86 nmol/mg Protein/hr, and his disease onset was 1 year 11 months old. It seemed that lower residual enzyme activities were associated with an earlier onset. However, in India report, patient 9 with undetectable ARSA activity had disease onset at 2 years 3 months old. Our limitation was that the case numbers were too small to draw a definitive conclusion.

In Asian countries, the number of reported cases with late infantile MLD is limited [13–19]. However, a great diversity of 22 different alleles of ARSA gene mutations was found (Table 2). Five novel mutations of ARSA gene were identified in our patients, including p.A316D, p.G303R, p.Q176X, p.R293X, and c.749 insGCGGGCCA. As the frequency of ARSA mutation alleles in normal alleles and pseudodeficiency alleles was equivalent [33, 34], it is possible that patients with late infantile MLD carry both disease-causing and pseudodeficiency alleles. Mutations whose effects might be exaggerated by the reduced synthesis of ARSA due to pseudodeficiency alleles are likely to be seen in late infantile MLD patients carrying both of them [33]. One of our 5 patients, patient 2, carried the ARSA gene mutations p.F249S and c.1344_1345 dupC and the ARSA pseudodeficiency allele p.N352S.

## Conclusions

In conclusion, patients with late infantile MLD exhibit a rapid and devastating clinical course. The initial neurological feature is gait disturbance followed by bed-ridden status within a couple of months to 2 years. Tigroid and leopard skin pattern of dysmyelination on brain MRI together with peripheral demyelination polyneuropathy serve as a clue indicating that evaluation of ARSA activity in leukocytes is warranted. Variable ARSA gene mutations could be found in patients with late infantile MLD in Asia, including p.W320X which was found in Taiwan and China, and p.G101V in Taiwan and Korea. An accurate diagnosis of late infantile MLD should be based on a combination of clinical features, biochemical analysis of low residual enzyme activity of ARSA in leukocytes, and pathognomonic ARSA gene mutations, in order to facilitate genetic counseling of family members and prenatal diagnosis.

## Abbreviations

AEP: Auditory evoked potential
ARSA: Arylsulfatase A
CNS: Central nervous system
CSF: Cerebrospinal fluid
DNA: Deoxyribonucleic acid
EEG: Electroencephalography
MLD: Metachromatic leukodystrophy
MRI: Magnetic resonance imaging
NCV: Nerve conduction velocity
PCR: Polymerase chain reaction
VEP: Visual evoked potential
